# Supplementary material for: Swimming behavior and hydrodynamics of the Chinese cavefish Sinocyclocheilus rhinocerous and a possible role of its head horn structure
Source: PLoS One. 2022 Jul 25;17(7):e0270967. doi: 10.1371/journal.pone.0270967 (PMC9312365; doi:10.1371/journal.pone.0270967)
Supplement: S1 Appendix — (DOCX) [file pone.0270967.s001.docx]

**S1 appendix. Training of ANN to Estimate Centroid locations**

Our fish motion capture and movement tracking system (Patent 202010478461.5) is based on an ANN (using U-Net [1]) that estimates the trajectory of the fish centroid. All top-view and side-view videos were converted into photo images. In Experiment 1, after lens correction, 220 top view photo images (Fig A1-A) and 220 side view images (Fig A1-B) were randomly selected. We then labeled the fish in each image (Figs. A1-A, B) and rotated the labeled results (Figs. A1-a, b) (90°, 180^o^ and 270^o^). The two 220-image datasets were expanded to two labeled-images datasets with 880 images each. For each of the 880 labeled-images datasets, 700 images were assigned to train the U-Net ANN model, and the other 180 images were used to validate the ANN model. The ANN model was trained using the labeled images with a stochastic gradient-descent implementation of Caffe [2]. For both datasets, the Dice coefficients for the validation after 100 epochs were over 0.99. Then the ANN model was used to compute the fish locations in the top and side view images (Fig A1-c and Fig A1-d, respectively).


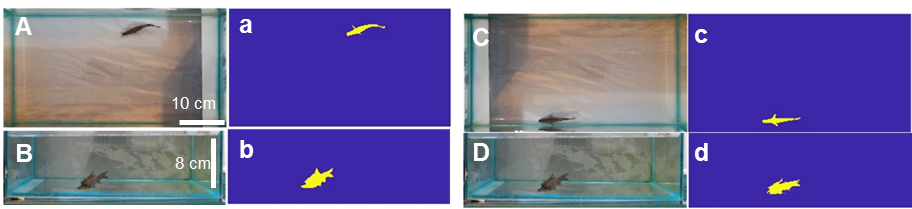


**Fig A1. Training and tracking processes**. Images shown were selected randomly from 220 top view photos and 220 side view photos: top view photos (A); side view photos (B). Corresponding images (a) and (b) were included in the training dataset, while images (c) and (d) illustrate fish tracking results of images (C) and (D) respectively; top (c) and side (d) view photos were then utilized to establish a 3d location.

The fish centroid coordinates ($x_{c}$, $y_{c}$, $z_{c}$) were then estimated as:

|  | $x_{c}=\frac{\sum_{x=1}^{N} \sum_{y=1}^{M} \sum_{Z=1}^{Q} x\times f(x,y,z)}{\sum_{x=1}^{N} \sum_{y=1}^{M} \sum_{Z=1}^{Q} f(x,y,z)}$, | (A1) |
| --- | --- | --- |
|  | $y_{c}=\frac{\sum_{x=1}^{N} \sum_{y=1}^{M} \sum_{Z=1}^{Q} y\times f(x,y,z)}{\sum_{x=1}^{N} \sum_{y=1}^{M} \sum_{Z=1}^{Q} f(x,y,z)}$, | (A2) |
|  | $z_{c}=\frac{\sum_{x=1}^{N} \sum_{y=1}^{M} \sum_{Z=1}^{Q} z\times f(x,y,z)}{\sum_{x=1}^{N} \sum_{y=1}^{M} \sum_{Z=1}^{Q} f(x,y,z)}$, | (A3) |

where *N*, *M*, and *Q* are the number of pixels along the *x*, *y*, and *z* axes, thus $x \epsilon[1,N]$, $y \epsilon[1,M]$, and $\text{z}\text{ ϵ [1,}Q\text{]}$, with

|  | $f\left( x,y,z \right)= \left\{ \begin{aligned} 1 , \mathrm{pixel} \left( x,y,z \right) on the fish body \\ 0, \mathrm{pixel} \left( x,y,z \right) not on the fish body \end{aligned} \right.$ ; | (A4) |
| --- | --- | --- |

$x_{c}$ and $y_{c}$ are obtained from a top view image, and $z_{c}$ from the corresponding side view image. This enables generation of the 3-D location ($x_{c}$, $y_{c}$, $z_{c}$), which is calculated 50 times per second.

**References in appendix**

1. Ronneberger O, Fischer P, Brox T. U-Net: convolutional networks for biomedical image segmentation. In: Navab N, Hornegger J, Wells W, Frangi A, editors. Medical Image Computing and Computer-Assisted Intervention – MICCAI. Cham: Springer; 2015. p. 1-8.
2. Jia Y, Shelhamer E, Donahue J, Karayev S, Long J, Girshick R, at al. Caffe: Convolutional architecture for fast feature embedding. MM '14: Proceedings of the 22^nd^ ACM international conference on Multimedia. 2014;675-678.
