# Supplementary material for: Swimming behavior and hydrodynamics of the Chinese cavefish Sinocyclocheilus rhinocerous and a possible role of its head horn structure
Source: PLoS One. 2022 Jul 25;17(7):e0270967. doi: 10.1371/journal.pone.0270967 (PMC9312365; doi:10.1371/journal.pone.0270967)
Supplement: S2 Appendix — (DOCX) [file pone.0270967.s002.docx]

**S2 appendix. Derivation of drag coefficient equation.**

The images from Experiment 2 were used to estimate the drag force during the coasting phase of swimming. Drag force on a swimming fish is related to the Reynolds number (Re) [1], given by

|  | $Re=\frac{\rho uBL}{\mu}$, | (A5) |
| --- | --- | --- |

where *ρ* and *μ* are the density and dynamic viscosity coefficient of water, respectively, $u$ is the fish’s swimming speed, and BL is its body length. Analysis of the recorded video in Experiment 2 yielded an average swimming speed of 1.57 cm/s, corresponding to Re ≃ 1,280. *S. rhinocerous* therefore swims in a flow regime with the drag force proportional to the square of the swimming speed ([2]:

|  | $F_{drag,coast}=-\frac{1}{2}C_{d,coast}\rho Au^{2}$, | (A6) |
| --- | --- | --- |

where $C_{d,coast}$ is the drag coefficient (during coasting phase), A is the surface area of the fish’s body, and $u$ is the swimming speed. At any time, t during the fish’s motion, the net force on the fish may be written as the sum of the thrust force generated by the fish to accelerate its body forward, the drag force, and the force needed to accelerate the added mass of fluid backward [3]:

|  | $F_{net}=ma= F_{thrust}+F_{drag}-k\rho Va$, | (A7) |
| --- | --- | --- |

where $a=\frac{du}{dt}$ is the acceleration. When the fish is coasting, $F_{thrust}=0$ and

${F_{drag}= F}_{drag,coast}$ , then

|  | $\left( m+k\rho V \right)\frac{\mathrm{du}}{\mathrm{dt}}= -\frac{1}{2}C_{d.coast}\rho Au^{2}$, | (A8) |
| --- | --- | --- |

where ($m+k\rho V$) is often called the “virtual mass” and *k* the “added mass coefficient” [5]. Integrating this expression (during the time in which the fish is coasting) yields Equation 5 in the paper for the reciprocal of the swimming speed, $\frac{1}{u(t)}$ . Equation 5 is consistent with [2]. A *k*-value of 0.045 based on a 1:1:6 ellipsoid has been used in zebrafish studies [2, 4], and, although *S. rhinocerous* is less streamlined than a zebrafish, this *k*-value is also adopted here. Fig 6A plots $\frac{1}{u(t)}$ *versus* time for one series of sequential images from Experiment 2.

**References in appendix**

1. Osse JWM., Drost MR. Hydrodynamics and mechanics of fish larvae. *Pol. Arch. Hydrobiol*. *1989;*36;455-465.
2. McHenry MJ, Lauder GV. The mechanical scaling of coasting in zebrafish (*Danio rerio*). Journal of Experimental Biology. *2005;*208:2289-2301.
3. Vogel S. *Life in Moving Fluids: The Physical Biology of Flow*, 2^nd^ Edn. Princeton, N.J.: Princeton University Press. 1994.
4. McHenry MJ, Lauder GV. Ontogeny of form and function: locomotor morphology and drag in zebraﬁsh (*Danio rerio*). Journal of Morphology. 2006;267:1099-1109.
