# Supplementary material for: Swimming behavior and hydrodynamics of the Chinese cavefish Sinocyclocheilus rhinocerous and a possible role of its head horn structure
Source: PLoS One. 2022 Jul 25;17(7):e0270967. doi: 10.1371/journal.pone.0270967 (PMC9312365; doi:10.1371/journal.pone.0270967)
Supplement: S3 Appendix — (DOCX) [file pone.0270967.s003.docx]

**S3 Appendix. Details of the CFD modeling of *S. rhinocerous* and *S. angustiporus* body shapes**

The 3D digital models of *S. rhinocerous* and *S. angustiporus* were obtained by applying scanning technology (Insight3 scanner produced by *Open Technologies srl* (Rezzato, Brescia, Italy) to specimens sampled from Luoping, Yunnan Province in Southwestern China. Fig 2B shows lateral views of the scanned models of *S. rhinocerous* and Fig 2D for *S. angustiporus*.

The 3-D digital models were loaded into ANSYS ICEM to build a mesh for CFD modeling. The control volume was assumed to be ten times that of the fish body size ($10BL\times10BW\times10BH$) to ensure that the flow field was fully developed. The mesh size outside of the fish boundary layer, *i.e*., more than 1 body size away from the fish, was 0.01m, and the mesh size within the boundary layer was 0.0025 m.

CFD simulations were run using the software Fluent (ANSYS, Inc.) under steady-state flow conditions passing along the static fish body at Re values from 900 to 10,000, representing the range of estimated Re values computed in Experiments 1 and 2. This corresponds to swimming velocities ranging from 1 to 10 cm/s. In the simulations, the fish’s head always faced toward the incoming flow, and the flow was assumed to be laminar. Velocity inlet, pressure outlet, and symmetry planes were assigned as boundaries. The physical parameters for the water flow were based on the cavefish specimens being sampled from caves at a notable elevation (~1400 m) with a water temperature of 18.5^o^C and were assigned as follows: gravitational acceleration *g* = 9.785 m/s^2^, density of water *ρ* = 991 kg/m^3^, water kinematic viscosity coefficient *μ* = 1.144 × 10^-3^ kg/(m·s), The solutions for pressure converged to a possible error of less than one part in ${10}^{-6}$.

**Table A1.** Sizes of 3D models of *S. rhinocerous* and *S. angustiporus*

| **Species** | **Length(cm)** | **Width(cm)** | **Height(cm)** | **Area(cm^2^)** | **Volume(cm^3^)** |
| --- | --- | --- | --- | --- | --- |
| *S. angustiporus* | 10.130 | 1.102 | 2.259 | 46.074 | 12.2103 |
| *S. rhinocerous* | 8.957 | 1.064 | 2.181 | 37.316 | 4.4162 |
