# Supplementary material for: Swimming behavior and hydrodynamics of the Chinese cavefish Sinocyclocheilus rhinocerous and a possible role of its head horn structure
Source: PLoS One. 2022 Jul 25;17(7):e0270967. doi: 10.1371/journal.pone.0270967 (PMC9312365; doi:10.1371/journal.pone.0270967)
Supplement: S3 File — (DOCX) [file pone.0270967.s006.docx]

These are the code introduction and tutorial, the “test_networks.py” is the main function for trainning, and the “ray_fish.py” is the main function for inferring the fish location. Both they are kept in “*\code\YDLL-master\src”. In order to using these code to track the fish location, you can operate as followings.

1. Software preparation

Install softaware Labelme, Anaconda and Visual Studio Code.

1. Data preparation

Choose around 200 photos randomly, and label the fish in using software Labelme. And then choose 80% labeled photos keeping in folder “Trainning”, and the other 20% in folder “Validating”.

1. Training the model

Run the function “test_networks.py” in Visual Studio Code.

1. Tracking the fish

Run the function “ray_fish.py” in Visual Studio Code. You can get the fish location in every photo.

1. Calculate the trajectory.
