# Supplementary material for: Swimming behavior and hydrodynamics of the Chinese cavefish Sinocyclocheilus rhinocerous and a possible role of its head horn structure
Source: PLoS One. 2022 Jul 25;17(7):e0270967. doi: 10.1371/journal.pone.0270967 (PMC9312365; doi:10.1371/journal.pone.0270967)
Supplement: Attachment — Submitted filename: Reply to Reviewers.docx [file pone.0270967.s008.docx]

**Reply to Reviewer #1**

Dear Dr. Yoshizawa,

Thank you very much for your time involved in reviewing the manuscript.

*Have the authors made all data underlying the findings in their manuscript fully available.*

**Response:**

Dr. Yoshizawa, We have upload all analysis data. But, We are sorry, the analysis code still can’t share in Githup now. For that we have accomplished the PIV analysis using an economic software. For behavior tracking, we have developed a tracking system, but we have applied a patent, if you have interest in it in future, you can search our patent 202010478461.5.

**Reply to Reviewer #2**

Dear reviewer,

**Comment 1:**

*“This is an improved version that is much more comprehensible than the previous one. However, there are still a number of minor, but also some more serious issues that need to be addressed and/or corrected.”*

**Response:**

Thank you very much for your time involved in reviewing the manuscript and your very encouraging comments on the merits. Your clear and detailed feedback help us to improve the present study much..

**Comment on title:**

***Title****: Just another suggestion. Simply “Swimming Behavior and Hydrodynamics of the Chinese cavefish, Sinocychlocheilus rhinoceros” or maybe “Swimming Behavior and Hydrodynamics of the Chinese cavefish, Sinocychlocheilus rhinoceros, and a possible role of its distinct head horn structure”*

**Response:**

**Title**: We have revised the title “Cavefish Hydrodynamics and Behaviors of the Chinese cavefish *Sinocyclocheilus rhinocerous* which possesses a head horn structure” into “Swimming Behavior and Hydrodynamics of the Chinese cavefish *Sinocyclocheilus rhinocerous* and a possible role of its head horn structure”

**Comments on abstract:**

*Abbreviations (CFD , PIV) are still present in the Abstract even though the definitions are spelled out. In most other journals this is not common. Abbreviations are normally given at the first site in the main text body where they are defined first, but not in an Abstract.*

*Line 25/26: “Particle Image Velocimetry”. Start each word with a capital letter.*

*Line 34: “sensing distance”. Do you mean sensing distance of the lateral line? If so, include this.*

*Line 36: Use past tense when reporting own data.*

*Line 36: If you include lateral line in line 34, then you should delete the text in parentheses here.*

*Line 44: The following comment applies to the statement that the sensitivity of sensing is increase in S.r. (i.e., here, in Methods, Results and Discussion). If I understand the paper correct, then there is greater pressure (pressure gradient) on the head of S. rhinoceros as compared to S. angustiporus. Thus, there is a stronger stimulus to this region in S.r. than in S.a. But this does not mean or show in any way that the neuromasts in this region have greater sensitivity. This can only be shown by comparing data from e.g., neuronal recordings from these neuromasts, or from observing neuromast motion in response to identical stimuli applied to both species. One could also do a histological survey of the neuromasts in that region and compare neuromast numbers, size, innervation etc. and from this conclude on sensitivity. The fact of a greater stimulus is no proof for greater sensitivity.*

**Response:**

Line 20, 23: We deleted the Abbreviations in abstract and gave the Abbreviations where they are defined first in the main text body, please see details in [Lines 20-21: We used a combined approach of laboratory observations and Computational Fluid Dynamic modeling]*.*

Line 25/26: We have revised "particle image velocimetry" into "Particle Image Velocimetry” in the abstract and main text.

Line 34: We have supplied the sensing distance information. Please see details in [Lines 22: *i.e.,* drag coefficients and lateral line sensing distance of *S. rhinocerous*.]

Line 36: We have revised the tense. Please see details in [Line 22, We used a combined approach of laboratory observations ; Line 35 The sensing distance was approximately 0.25 body lengths].

Line 36: We have deleted the text in parentheses.

Line 44: We have revised the sensitivity into the lateral line stimulus, please see details in [Lines 40-42:Results support the hypothesis that the conspicuous cranial morphology of *S. rhinocerous* may lead to greater the lateral line stimulus compared to fish that don’t possess such morphology. ]

**Comments on introduction:**

*The beginning of the Introduction is much clearer now due its shortness. However, the end of the Introduction can still be streamlined further. Presently there are numerous methodical aspects presented at the end of the Introduction, that should be reported in the Methods part (see comments below).*

*Line 85 and elsewhere in manuscript: There is no need any more to refer to the field survey (except at one site in the Discussion)*

*Line 102 ff: I appreciate the description of the lateral line morphology of S.r.. But (and perhaps I made this comment before), you nonetheless need to give the original reference here or instead show your own data. I checked references 13-15 but S.r. is not mentioned in any of these references.*

*Line 130: I suggest a line break here after (NACA0013).*

*Line 139: “increase the sensitivity”. Same comment as in Abstract line 44. There is a greater stimulus on the head but this is no proof of greater sensitivity of the receptors (see also comment above and elsewhere in this list).*

*Line 150 and also 136: You tend to repeat “This paper” or “In this paper”. Try to avoid these repetitions. E.g., here you can simply delete it. Also, lines 150-152 are more or less the same as lines 113-120. Go through the manuscript and remove such redundancies wherever they occur.*

*Lines 152 ff: I suggest to keep the end of the Introduction as straight-forward as possible by moving all the methodical details to the Method section. For example, one could simply say: “In this paper, a combined approach of laboratory observations including fish tracking, PIV and CFD modeling was used to characterize the swimming behavior and hydrodynamics of S. rhinocerous. The results of our data analysis suggests that …” All the rest, the pros and cons and the good and bads of your methods can go into the Methods section.*

**Response**

Line 85: We have deleted most field survey description in Introduction and in Methods and Materials.

Line 102: Yang J., 2016 is the only paper which reported the superficial neuromasts distribution of *S. rhinocerous*, but they only showed the superficial neuromasts on the head, please see [Yang j., Chen X., Bai, C., Fang, D., Qiu, Y. The *Sinocyclocheilus* cavefish genome provides insights into cave adaptation.]. In this study, We have carried stain experiments on *S. rhinocerous to obtain* the whole surperficial neuromasts. We have separates Fig 8, and removed the stain results Fig 8A here to show the extensive array of superficial neuromasts that extend from immediately behind the head until near the base of the tail. We used the references 13-15 to help explain the morphology of lateral line system (including superficial neuromasts and canal neuramasts which are not shown here).

**Fig 2. Superficial neuromasts distribution of *S. rhinocerous.*** The yellow points are the superficial neuromasts and obtained by DASPEI stain, the upper two photographs show a dorsal view and the lower two show a side view of the *S. rhinocerous*;

Line 139: We have revised the “sensitivity” into “lateral line stimulus” in the abstract and the whole main text.

Line 150 and also 136: We have deleted “in this paper”. And try to delete the similar repetitions.

Lines 152: We have removed the laboratory description into Methods and Materials to simple the introduction, please see details in [Lines 134-150: A combined approach of laboratory observations and CFD modeling was applied. By combining the laboratory measurements with CFD modeling, a more complete depiction of the fluid dynamics, pressure distribution, and shear distribution along the fish body can be obtained. CFD modeling (utilizing ANSYS software) was performed on digital models of the troglobite *S. rhinocerous* and, for comparison, the troglophile *S. angustiporus*. These models were generated from scans of preserved specimens. This is in contrast to earlier studies that approximated the fish’s body by a few chosen basic, axially-symmetric (along the anterior to posterior axis) geometric shapes [17, 40, 41]. Windsor *et al.* [40, 41] used a (two-) three-dimensional NACA 0013 aerofoil to obtain a (2-D) 3-D model for the surface of the fish’s body. In the present study, laboratory and simulation results are combined to chart 3-D trajectories and map out velocity fields, and these are employed to elucidate the fish’s swimming behavior. Mapping out the flow field around the fish enables an estimation of drag coefficients and sensing distances. The discussion and conclusion contain a justification of the assertion that the unique morphological features of troglobites such as *S. rhinocerous* influence neuromast perception, and describe some possible next steps.]

**Comments on Materials and methods:**

*Line 187: “field survey”. Not needed any more (see above).*

*Line 202: Must be specimen, not speciemen.*

*Line 206: “scanned to obtain digital modeling”. Do you mean “scanned for imaging and subsequent digital modeling”?*

*Line 211-215: It is too bad that behavioral data were collected on only a single fish. Results probably differ quite a bit when fish of different size were used. It would good to have a feeling for this variability.*

*Line 215: Should be specimen, not specimens since all experiments were done on a single fish.*

*Line 215: “not affect behavior”. What exactly is meant here? Which aspect of behavior? How do you know? How did you control for possible effects?*

*Line 225ff: Here you could include all the details of the tracking system.*

*Line 232: I guess this is length x width x height? What was the water level? Also 8 cm? Or lower? This would then essentially be a 2D-pkane in which the fish was moving.*

*Line 236ff: Did camera 3 really have a frame rate of only 10 Hz? This then limits you PIV analysis to 10 Hz.*

*Lines 241ff: In this section you can include all the details on the PIV system.*

*Line 251: “A laser pulse operated continuously”. This sentence is ambiguous. Be more specific. Also, the information provided here is rather limited. Did you use a PW or a CW laser? I assume it was a PW laser that was turned on for the entire recording session? Please make this clear. Also, what laser model did you use? What was the wavelength (IR, red, green)? How did you produce the laser sheet? I assume you used a dispersion lens? What was your level of analysis (pixel size, interrogation window etc.)?*

*Line 261: “50 times per second”. If camera 3 was operating at 10 Hz, then you cannot be faster than this.*

*Line 308ff: I am not sure if I understand this correctly. You measure an abrupt change in energy across the laser plane caused by the approaching fish (Fig. 7A). This depends on swimming velocity. The faster the fish, the earlier you detect this. In other words, the faster the fish, the further the fish is away from the laser plane when you detect the disturbance (Fig. 7B, fish, most likely the bow wake, increases with swimming speed? But I still do not understand why this translates into sensing distance for the fish lateral line system? Please help the reader here. Do you want to say that the fish detects its own bow wake? But then why does it need 0,2 BL for this? Are there no neuromasts near the tip of the snout?*

*Line 317: It would be helpful if you make clear that this was a simulated flow that was continuously on and not a real flow in a flow channel. The reader may have forgotten or overlooked this here.*

*Line 352: “2% of body length”. Why do you make such assumptions? From your writing further below, I infer that you do have the real data on canal pore spacing. So why not use the real data?*

*Line 358: reference 50 is a book chapter by Jorgensen. While Jorgensen does refer to DASPEI stain as a method, I have not found any reference to S. rhinoceros in that chapter. As requested further up, I ask for the original reference or show you own data.*

**Respose**

Line 187: We have deleted most descriptions on field survey in Methods and Materials, please see details in Lines 153 and 158-160.

Line 202: We have revised “speciemen” into “specimen”, please see details in Line 165.

Line 206: We have revised it, please see details in [Lines 175-177: The specimens that died during the transportation and maintenance at the facility were preserved in 75% alcohol solution, and the biggest one sampled was scanned for imaging and subsequent digital modeling (Figs 1B and 1D)]

Line 211-215: In this study, the experiment measurement conclusions of the swimming speed range and near-wall-following behaviors will not be changed when increase the fishes in experiment. And the conclusions on simulations will not be changed. But the the average swimming speed will vary. So, the next step, further experimental analyses will try to involve a larger number of test specimens to check the conclusions in this study and enable a closer examination of variability among conspecific specimens. Luckily, now, the artificial propagation of *S. rhinoceros* has been successful last year, so we have opportunity to observe more behaviors in future.

Line 215: We have revised the “specimens” into “specimen”.

Line215:We have revised the “not affect behavior” into “*S. rhinoceros* has no light reaction behavior”. “Not affect behavior” means that there is no light reaction for *S. rhinocerous*. In 2016, we have a undergraduate student (Chen H., 2016) have done a experiment to test the light reaction behavior. Please see details in [Line 178: ambient light (*S. rhinocerous* has no light reaction]

Line 225: We have moved the details of tacking system and PIV in introduction here, please see details in [Lines 188-197: The laboratory research consisted of observations of fish swimming in an aquarium. This utilized a motion capture and tracking system coupled to a Particle Image Velocimetry (PIV) system to map out water-velocity fields. A tracking system with an Artificial neural network (ANN), Convolutional Networks for Biomedical Image Segmentation (U-Net) [44] was utilized to generate accurate trajectories (Patent 202010478461.5). A PIV system was also used to obtain qualitative and quantitative descriptions of the flow fields around the fish body during swimming. Such flow fields can be used to estimate the energetic costs of swimming behaviors [46], pressure distributions [40, 41], thrust impulse [47, 48], and active drag [49].] And for more details you can see supplementary S1.

Line 232: The tank height is 20cm, in this paper we only reported the water volume, so the water level is 8cm.

Line 236ff: Yes in the PIV system, the frequency of camera3 is only 10Hz. When only tracking the trajectory (Using camera1 and camera2), we can obtain the data in 50Hz. But when combining the PIV system (Camera 3), we can only obtain the data in 10HZ.

Line 241ff: We have moved the details of tacking system and PIV in introduction here, please see details in [Lines 188-197].

Line 251:I have supplied the details on PIV, please see details in [Line 222-226: A PW laser was turnen on, PIV model, blue light, sheet laser, 32×32 pixel size were selected (Lifang Technique company, China) with the fish crossing the laser plane multiple times. The program MicroVec V2.0 System (Tianjin University) was used to generate 2-D velocity fields.]

Line 261: The frequency of the camera1 and camera2 are 50Hz, when tracking only, we can track 50 locations per second at most. Only when combing with camera3, we will track 10 locations per second.

Line 308ff: Yes, in the PIV system, the frequency of camera3 is only 10Hz. When only tracking the trajectory (Using camera1 and camera2), we can obtain the data in 50Hz. But when combining with the PIV system (Camera 3), we can only obtain the data in 10HZ.

Line 317: We have supplied the details in [Lines 290-293: For model simulations, this was a simulated flow that was continuously on and not a real flow in a flow channel, and the fish’s body was aligned such that the head faced the incoming flow. This minimized effects due to the tail and was taken to represent cruising behavior.]

Line 352: Like previous question in Line 102, we have not figured out the details on canla neuromasts distribution, and in order to compare the results with previous studies on Mexican cave characin, *Astyanax mexicanus* (Windsor et al., 2010a), we assumed that canal pores spaces at 2% body length (the same with them).

Line 358: We referenced Jorgensen [58] here to show the stain methods.

**Comment on results:**

*Line 369: “more randomly”. There is no comparative to random. Also, how did you test for randomness?*

*Lines 368ff: You analyzed the data from the 3rd hour but you show the data from hours 1 and 2. Where are the data from the 3rd hour? Show!*

*Line 403: must be “shows”*

*Line 403ff: Do I understand correctly that Fig. 6A show data from one example? So what is the drag coefficient that results from this example?*

*Line 407: Can’t be the same Re value because data points in figure are not aligned on the X-axis.*

*Line 408: “consistent with that obtained from Experiment 2”*

*Line 409: “tended”. Why tended? I think it is very clear from the figure that they did decrease.*

*Line 410/411: “were similar to, but consistently higher than those …”*

*Line 422: “indicates”*

*Line 428 and subsequent paragraph: sensing distance. See my questions above.*

*Line 429ff: The description of the data still does not match the data shown. The text reads as if the velocity in stage 3 is similar to that in stage 1, which is evidently not the case. During stage 3, the velocity is still greater than during stage 1, and this should be described as such. The figure does not show if velocity decreased eventually to values similar to this in stage 2, but you can say this but have to make it clear.*

*Line 443ff: What part of the 40x8x8 cm tank is shown in the lower part of the figure? Is this a vertical or a horizontal laser plane? Can you draw the outlines of the fish into the lower figures?*

*Line 450: “Lateral stimulus”. Do you mean “Lateral line stimulus”?*

*Line 451 and figures 8 and 9: Apparently, part A of Figure 8 shows a DASPEI stain. You need to say this somewhere in the text or legend. You also need to indicate that the upper two photographs show a dorsal view and the lower two show a side view of the fish. By the way, are these from the same fish or from two different fish? Also, separate the upper two photographs as you did the lower two.I assume the scale bar of 1 cm in the upper right photograph refers to all 4 photographs. This means that the upper magnification (dotted rectangle) is ca. 5x5 mm and the lower one ca. 3x2 mm.Now, by looking at the magnifications (left photographs in 7A), I do see lines of dots extending from left to right on the head and from dorsal to ventral on the side. However, I do not see something like a trunk lateral line canal. Moreover, each line in the lower figure has at least 10, if not more dots, i.e., at least 5 dots per 2 mm. This all makes me assume that these are not canal neuromasts that are stained here, but rather superficial neuromasts. In any case, the spacing is much greater than the 2 mm pore distance assumed for the CFD analysis that is shownin part B of Figure 8. If the stained neuromasts in Fig. 8A are indeed superficial neuromasts, then showing this is not helpful. Instead, the reader needs to see a stain of the canals, the canal pores and/or the canal neuromasts. To show the canals and the pores, one could simply fill the canal with methylene blue to visualize its course. The pores and the canal neuromasts normally also are stained in a DASPEI stain, but in Fig. 8A I do not see them. But it is necessary for your conclusions to show that indeed there are canal neuromasts in that region.*

*Line 482, figure legend Fig. 9: “Lateral line stimulus”? You may also point out here, that the Y-axis is plotted reversed (from negative to positive values).Figures 8-9: At some point you should explicitly write, how you come from the data shown in Fig. 8B to the curves shown in Fig. 9A. I assume that you plotted normalized pressure along the putative canal neuromasts (dots in Fig. 8B)? But you do not explain this. Help the reader here.Figure 9: I compared that curves in Fig. 9A with those in Fig. 7 by Windsor et al (2010a). While the blue curve seems to be the curve from the Hassan-data. The green curve seems to be a curve that is derived from a NACA airfoil. The actual 2D fish data look different. Check red curve in Fig. 7 by Windsor et al). It actually does have two negative peaks but the positive peak in between is very much smaller than the one here in S. rhinoceros.Also, I recommend to label all curves with the species name (A. mexicanus in both cases) and give the respective references in parentheses).*

**Response**

Line 369: We have deleted the more, please see details in [Line 343-145: During Experiment 1, *S. rhinocerous* tended to swim near a wall during the first hour and then swim randomly throughout the tank during the second hour (Fig 5A).] In the last version, when we say more randomly, we compared the trajectory in the second hour with the first hour.

Line 368: No, we analyzed the data in the first two hours rather than the third hour in behavior studies for that the trajectory in the third hour is similar to that in the second hour. Please see details in [Lines 201-202: It was found that the fish behaved differently in the first two hours and became stable in the third hour, thus we analyzed the swimming behavior during the first two hours.]

Line 403: We have revised the “showed” into “shows”, please see details in [Line 372: Fig 7A shows a drag coefficient estimation example]

Line 403ff: Fig 7A is an example and the drag coefficient of this example can be derived from the slope coefficient, the drag coefficient in this example is 0.074.

Line 407: The Re in experiment is estimated by the average swimming speed, so it is fixed.

Line 408: We have revised, please see details in [Lines 376-378: For the same Re value, the $C_{d,coast}$estimate obtained via Eqn. 7 from the CFD simulations for *S. rhinocerous* was generally consistent with that obtained from Experiment 2.]

Line 409: We have deleted the tended to, please see details in [Line 378: The simulation estimate for $C_{d,coast}$decreased with increasing Re ].

Line 410/411: We have revised, please see details in [lines 375-376: The CFD-estimated values for $C_{d,coast}$for *S. rhinocerous* were similar to, but consistently slightly higher than those for *S. angustiporus*.]

Line 422: We have revised the “indicated” into “indicates”in the legend of Fig 7.

Line 428: We have supplied the lateral line to the sensing distance.

Line 429: We have revised it, please see details in [Line 407-410: Finally, as the fish exited the laser plane, $E_{L}$ decreased and the water in the laser plane gradually resumed its initial undisturbed if the *S. rhinocerous* swam far away enough to the laser plane and the water velocity decreased eventually to values similar to this in stage 1].

Line 443ff: the Fig. 8C shows around 8×6cm plane, and we have tried to draw the outline of the fish, but it only showed an irregular ellipse and is not able to help readers to distinguish fish, so we give up.

Line 450: We have revised the “lateral stimulus (CFD)” into “lateral line stimulus (CFD)”.

Line 451: We have Separated the Fig 8 into Fig 2 and Fig 9. In Fig 9, the canal pores were assumed to be spaced at 2% body length (BL) intervals as illustrated on the scanned model cavefish body. This pore interval is consistent with an earlier study of Mexican cave characins (Windsor et al., 2010a).

Line 482: we have supplied the “Lateral line stimulus” in Fig 10B. And we have labeled the “the Y-axis is plotted reversed” in the legend of Fig 10. We have explained that Fig 10A shows the normalized pressure along the putative canal neuromasts (dots in Fig. 9) in text, please see details in [Lines 440-441: Fig 10A shows the normalized pressure along the putative canal neuromasts in Fig 9.].The green curve is derived from NACA0013 3D which was used to represent *Astyanax Mexicanus* (Windsor et al., 2010a), we have labeled it in Fig 10.

Fig 10. **Lateral line stimulus estimation.** CFD results showing (A) the normalized pressure, or *C_p_*, and (B) ${\Delta C}_{p}$ values along the assumed line of canal neuromasts for the simulations. Results extracted from earlier studies are also included. The Y-axis is plotted reversed.

**Comments on discussion:**

*Line 495-496: The sentence reads odd. Maybe you need to shorten this to “Understanding the …. may help to discern …”*

*Line 509: Unclear. Do you mean that the wall-following phase is the exploratory phase?*

*Line 525: Delete text in parenthesis. You explain the methods a few lines further below.*

*Line 545-546: slow swimming may improve sensory capabilities. Why? Give a rational or a logic why this is so.*

*Line 553: “It is conjectured“. Do you want to say “We therefore hypothesize…”?*

*Line 563: pressure fields matched the locations of the canal pores. Unclear description. Be more explicit. What exactly do you mean?*

*Line 573: “It is conjectured“. Do you want to say “We propose that…”? After all, the data do not and cannot show this.*

*Line 607: Why should this not have a similar function. If the form is similar to the form in S.r., then one would expect similar hydrodynamic effects. And as you hypothesize yourself, better sensing may lead to increased fitness, even if thus a trait only found in males.*

*Lines 656ff: I really think that the future plans can be omitted. By all due respect for your research, but it is really not important for the present paper what you want to do next. If these plans contain arguments relevant for the interpretation of the present data, then these arguments can be discussed in those sections of the Discussion where they are appropriate. If this future research is essential for the data, then maybe one should perform this research now and include it in the paper.*

**Response**

Line 495-496: We have revised this sentence, please see details in [lines 457-459: Understanding the swimming and sensory capabilities of troglobite cavefish may help discern micro-habitat preferences and dispersal patterns of the cavefish in their underground environments]

Line 509: Yes, the near-wall-following phase is the exploratory phase in new environment.

Line 525: We have deleted the text in parenthesis.

Lines 545-546:Slow swimming didn’t improve sensory capabilities but will help save energy to help to adapt the oligotrophic subterranean environment.

Line 563: We have revised this sentence, please see details in [Lines 521-522: The simulated pressure value in the location of the canal pores in Fig 9 for *S. rhinocerous* and *S. angustiporus* was extracted.]

Line 573: “it is conjectured”means “we proposed that”, here, for that the lateral line plays key sensing role in near-wall following behavior.

Line 607: I agree with you, the head horn structure of female *Kurtus gulliveri* may also have the same function on increasing the lateral line stimulus. But for the *Kurtus gulliveri* head horn structure, this function is not neccessary. We have adjusted the writting, please see details in [Lines 555-558: There are a few non-hypogean fish species that also have a head-horn. For instance, Indo-Pacific surgeonfishes of the genus Naso (Family *Acanthuridae*); however, it is unlikely that the same function for Naso head-horn is neccessary. Another example is the nurseryfishes of Australia and Asia (Family *Kurtidae; Kurtus gulliveri* and *Kurtus indicus*, respectively)]

Line 656ff: Thank your suggestion, and we agree that the future plans part are not neccessary for the present papers. But we hope to keep this part to help discuss the key advances of this study and the next step to improve this work.
