# Supplementary material for: Swimming behavior and hydrodynamics of the Chinese cavefish Sinocyclocheilus rhinocerous and a possible role of its head horn structure
Source: PLoS One. 2022 Jul 25;17(7):e0270967. doi: 10.1371/journal.pone.0270967 (PMC9312365; doi:10.1371/journal.pone.0270967)
Supplement: Attachment — Submitted filename: Reply to Reviewers.docx [file pone.0270967.s009.docx]

**Reply to Reviewer #1**

Dear Dr. Yoshizawa,

Thank you for your comments on the manuscript.

*Have the authors made all data underlying the findings in their manuscript fully available.*

**Response:**

We have uploaded all analysis data and the Artificial Neural Network code used to track the fish movement, including a tutorial on how to use the code. If you have any questions when using it, you can contact us with kairay04@163.com.

**Reply to Reviewer #2**

**Comment 1:**

*“This is an improved version that is much more comprehensible than the previous one. However, there are still a number of minor, but also some more serious issues that need to be addressed and/or corrected.”*

**Response:**

Thank you very much for your time and effort in reviewing our manuscript. We document our revisions in the replies to the reviewer’s comments below. We label each comment with the line number referenced by the reviewer. Where appropriate, we refer to the new line numbers where our revisions can be found.

**Comment on title:**

***Title****: Just another suggestion. Simply “Swimming Behavior and Hydrodynamics of the Chinese cavefish, Sinocychlocheilus rhinoceros” or maybe “Swimming Behavior and Hydrodynamics of the Chinese cavefish, Sinocychlocheilus rhinoceros, and a possible role of its distinct head horn structure”*

**Response:**

**Title**: We have revised the title as suggested to “Swimming Behavior and Hydrodynamics of the Chinese cavefish *Sinocyclocheilus rhinocerous* and a possible role of its head horn structure”.

**Comments on abstract:**

*Abbreviations (CFD, PIV) are still present in the Abstract even though the definitions are spelled out. In most other journals this is not common. Abbreviations are normally given at the first site in the main text body where they are defined first, but not in an Abstract.*

*Line 25/26: “Particle Image Velocimetry”. Start each word with a capital letter.*

*Line 34: “sensing distance”. Do you mean sensing distance of the lateral line? If so, include this.*

*Line 36: Use past tense when reporting own data.*

*Line 36: If you include lateral line in line 34, then you should delete the text in parentheses here.*

*Line 44: The following comment applies to the statement that the sensitivity of sensing is increase in S.r. (i.e., here, in Methods, Results and Discussion). If I understand the paper correct, then there is greater pressure (pressure gradient) on the head of S. rhinoceros as compared to S. angustiporus. Thus, there is a stronger stimulus to this region in S.r. than in S.a. But this does not mean or show in any way that the neuromasts in this region have greater sensitivity. This can only be shown by comparing data from e.g., neuronal recordings from these neuromasts, or from observing neuromast motion in response to identical stimuli applied to both species. One could also do a histological survey of the neuromasts in that region and compare neuromast numbers, size, innervation etc. and from this conclude on sensitivity. The fact of a greater stimulus is no proof for greater sensitivity.*

**Response:**

We have deleted the abbreviations in abstract and defined the abbreviations where they are defined first in the main text body.

Line 25/26: We have revised "particle image velocimetry" into "Particle Image Velocimetry” in the abstract and main text.

Line 34: We have added the sensing distance information to the text. The added text now refers specifically to “drag coefficients and lateral line sensing distance of *S. rhinocerous*” and that the distance was approximately 0.25 body lengths.

Line 36: As suggested, with the additional text we deleted the text in parentheses.

Lines 44: We have eliminated the term “sensitivity” and now state the result in terms of a greater stimulus of the lateral line. This has been done for all occurrences in the manuscript.

**Comments on introduction:**

*The beginning of the Introduction is much clearer now due its shortness. However, the end of the Introduction can still be streamlined further. Presently there are numerous methodical aspects presented at the end of the Introduction, that should be reported in the Methods part (see comments below).*

*Line 85 and elsewhere in manuscript: There is no need any more to refer to the field survey (except at one site in the Discussion)*

*Line 102 ff: I appreciate the description of the lateral line morphology of S.r.. But (and perhaps I made this comment before), you nonetheless need to give the original reference here or instead show your own data. I checked references 13-15 but S.r. is not mentioned in any of these references.*

*Line 130: I suggest a line break here after (NACA0013).*

*Line 139: “increase the sensitivity”. Same comment as in Abstract line 44. There is a greater stimulus on the head but this is no proof of greater sensitivity of the receptors (see also comment above and elsewhere in this list).*

*Line 150 and also 136: You tend to repeat “This paper” or “In this paper”. Try to avoid these repetitions. E.g., here you can simply delete it. Also, lines 150-152 are more or less the same as lines 113-120. Go through the manuscript and remove such redundancies wherever they occur.*

*Lines 152 ff: I suggest to keep the end of the Introduction as straight-forward as possible by moving all the methodical details to the Method section. For example, one could simply say: “In this paper, a combined approach of laboratory observations including fish tracking, PIV and CFD modeling was used to characterize the swimming behavior and hydrodynamics of S. rhinocerous. The results of our data analysis suggests that …” All the rest, the pros and cons and the good and bads of your methods can go into the Methods section.*

**Response:**

Line 85: We have removed the text that refers to the field surveys in the Introduction and in the Methods and Materials.

Line 102: We added a new Figure 2 that resulted from splitting old Figure 8 into two figures. This enabled us to more clearly show the extensive array of superficial neuromasts that extend from immediately behind the head until near the base of the tail. We used the references 13-15 to help explain the morphology of lateral line system (including superficial neuromasts and canal neuramasts which are not shown here). Yang et al. (2016) is the only paper we are aware of that reported the distribution of superficial neuromasts of *S. rhinocerous*, but they only showed the superficial neuromasts on the head, please see [Yang J., Chen X., Bai, C., Fang, D., Qiu, Y. The *Sinocyclocheilus* cavefish genome provides insights into cave adaptation.]. In our study, we have carried stain experiments on *S. rhinocerous* to obtain the entire distribution of surperficial neuromasts.

**Fig 2. Superficial neuromasts distribution of *S. rhinocerous.*** The yellow dots are the superficial neuromasts and obtained by DASPEI stain. The upper two photographs show a dorsal view and the lower two show a side view of the *S. rhinocerous*.

Line 139: We have revised the “sensitivity” to “lateral line stimulus” in the abstract and the whole main text.

Line 150 and also 136: We have replaced “In this paper” throughout the text with other phrases, such as “We..” and “Our…”

Lines 152: We have removed the laboratory description from the end of the Introduction into the Methods and Materials to simplify the Introduction. The moved paragraph begins with “The laboratory research consisted of observations of fish swimming in an aquarium. This utilized,… pressure distributions [40, 41], thrust impulse [47, 48], and active drag [49]”. Please see details in Lines 189-198.

**Comments on Materials and methods:**

*Line 187: “field survey”. Not needed any more (see above).*

*Line 202: Must be specimen, not speciemen.*

*Line 206: “scanned to obtain digital modeling”. Do you mean “scanned for imaging and subsequent digital modeling”?*

*Line 211-215: It is too bad that behavioral data were collected on only a single fish. Results probably differ quite a bit when fish of different size were used. It would good to have a feeling for this variability.*

*Line 215: Should be specimen, not specimens since all experiments were done on a single fish.*

*Line 215: “not affect behavior”. What exactly is meant here? Which aspect of behavior? How do you know? How did you control for possible effects?*

*Line 225ff: Here you could include all the details of the tracking system.*

*Line 232: I guess this is length x width x height? What was the water level? Also 8 cm? Or lower? This would then essentially be a 2D-pkane in which the fish was moving.*

*Line 236ff: Did camera 3 really have a frame rate of only 10 Hz? This then limits you PIV analysis to 10 Hz.*

*Lines 241ff: In this section you can include all the details on the PIV system.*

*Line 251: “A laser pulse operated continuously”. This sentence is ambiguous. Be more specific. Also, the information provided here is rather limited. Did you use a PW or a CW laser? I assume it was a PW laser that was turned on for the entire recording session? Please make this clear. Also, what laser model did you use? What was the wavelength (IR, red, green)? How did you produce the laser sheet? I assume you used a dispersion lens? What was your level of analysis (pixel size, interrogation window etc.)?*

*Line 261: “50 times per second”. If camera 3 was operating at 10 Hz, then you cannot be faster than this.*

*Line 308ff: I am not sure if I understand this correctly. You measure an abrupt change in energy across the laser plane caused by the approaching fish (Fig. 7A). This depends on swimming velocity. The faster the fish, the earlier you detect this. In other words, the faster the fish, the further the fish is away from the laser plane when you detect the disturbance (Fig. 7B, fish, most likely the bow wake, increases with swimming speed? But I still do not understand why this translates into sensing distance for the fish lateral line system? Please help the reader here. Do you want to say that the fish detects its own bow wake? But then why does it need 0,2 BL for this? Are there no neuromasts near the tip of the snout?*

*Line 317: It would be helpful if you make clear that this was a simulated flow that was continuously on and not a real flow in a flow channel. The reader may have forgotten or overlooked this here.*

*Line 352: “2% of body length”. Why do you make such assumptions? From your writing further below, I infer that you do have the real data on canal pore spacing. So why not use the real data?*

*Line 358: reference 50 is a book chapter by Jorgensen. While Jorgensen does refer to DASPEI stain as a method, I have not found any reference to S. rhinoceros in that chapter. As requested further up, I ask for the original reference or show you own data.*

**Response:**

Line 187: We have deleted most descriptions about the field survey in the Methods and Materials, please see details in Lines 154 and 159-160.

Line 202: We have revised “speciemen” into “specimen”. please see details in Line 166.

Line 206: We have revised the text as suggested, please see details in [Lines 168-170] The new text is “The specimens that died during the transportation and maintenance at the facility were preserved in 75% alcohol solution, and the biggest one sampled was scanned for imaging and subsequent digital modeling (Figs 1B and 1D)]”

Line 211-215: We have discussed the possible limitation that using one fish in the section on Future Plans. Please see details in [Lines 612-615]. The new text is “Our results are dependent on one individual fish that can limit the robustness of our results. While we are confident the behavior and morphology are representative, individuals will vary in other aspects (e.g., swimming speed) that could affect the specifics of our results.”

Line 215: We have revised the “specimens” into “specimen”.

Line215: We have revised the “not affect behavior” into “*S. rhinoceros* does not react to light”.

Line 225, 241ff: We have moved the paragraph about details of tacking system and PIV from the Introduction to here. Please see details in Lines 189-198.

Line 232: The water-volume tank is 40 × 25 × 8 (cm) (length × width × height). Please see details in Lines 204-205.

Line 236ff, Line 261ff, 308ff: In the PIV system, the frequency of camera3 is only 10Hz. When only tracking the movement (Using camera1 and camera2), we can obtain the data in 50Hz. But when combining the PIV system (Camera 3), we can only obtain the data in 10HZ. We added that the data collected was at 50HZ for Experiment 1 that used cameras 1 and 2 but 10HZ for Experiment 2 because cameras 3 was included as part of the PIV.

Line 251: I have added more details about the PIV, the new text [Line 225-230] is “A PW laser was turned on, PIV model, blue light, sheet laser, 32×32 pixel size were selected (Lifang Technique company, China) with the fish crossing the laser plane multiple times….” The program MicroVec V2.0 System (Tianjin University) was used to generate 2-D velocity fields.

Line 317: We have added the reminder about the simulations [Lines 294-296] We added “… this was a simulated flow that was continuously on and not a real flow in a flow channel, and …”

Line 352: In order to compare the results with previous studies on Mexican cave characin, *Astyanax mexicanus* (Windsor et al., 2010a), we assumed that canal pores spaces at 2% body length (the same with them). We added a new sentence [Lines 335-337]: “More quantitative evaluation of canal pore spacing to compare to other species using our specimen was not supportable.”

Line 358: We referenced Jorgensen [58] here to show the stain methods. [Line 339] We added text that explains Jorgensen discusses the stain but not specifically *S. rhinocerous.*

**Comment on results:**

*Line 369: “more randomly”. There is no comparative to random. Also, how did you test for randomness?*

*Lines 368ff: You analyzed the data from the 3rd hour but you show the data from hours 1 and 2. Where are the data from the 3rd hour? Show!*

*Line 403: must be “shows”*

*Line 403ff: Do I understand correctly that Fig. 6A show data from one example? So what is the drag coefficient that results from this example?*

*Line 407: Cant be the same Re value because data points in figure are not aligned on the X-axis.*

*Line 408: “consistent with that obtained from Experiment 2”*

*Line 409: “tended”. Why tended? I think it is very clear from the figure that they did decrease.*

*Line 410/411: “were similar to, but consistently higher than those … ”*

*Line 422: “indicates”*

*Line 428 and subsequent paragraph: sensing distance. See my questions above.*

*Line 429ff: The description of the data still does not match the data shown. The text reads as if the velocity in stage 3 is similar to that in stage 1, which is evidently not the case. During stage 3, the velocity is still greater than during stage 1, and this should be described as such. The figure does not show if velocity decreased eventually to values similar to this in stage 2, but you can say this but have to make it clear.*

*Line 443ff: What part of the 40x8x8 cm tank is shown in the lower part of the figure? Is this a vertical or a horizontal laser plane? Can you draw the outlines of the fish into the lower figures?*

*Line 450: “Lateral stimulus”. Do you mean “Lateral line stimulus”?*

*Line 451 and figures 8 and 9: Apparently, part A of Figure 8 shows a DASPEI stain. You need to say this somewhere in the text or legend. You also need to indicate that the upper two photographs show a dorsal view and the lower two show a side view of the fish. By the way, are these from the same fish or from two different fish? Also, separate the upper two photographs as you did the lower two.I assume the scale bar of 1 cm in the upper right photograph refers to all 4 photographs. This means that the upper magnification (dotted rectangle) is ca. 5x5 mm and the lower one ca. 3x2 mm.Now, by looking at the magnifications (left photographs in 7A), I do see lines of dots extending from left to right on the head and from dorsal to ventral on the side. However, I do not see something like a trunk lateral line canal. Moreover, each line in the lower figure has at least 10, if not more dots, i.e., at least 5 dots per 2 mm. This all makes me assume that these are not canal neuromasts that are stained here, but rather superficial neuromasts. In any case, the spacing is much greater than the 2 mm pore distance assumed for the CFD analysis that is shownin part B of Figure 8. If the stained neuromasts in Fig. 8A are indeed superficial neuromasts, then showing this is not helpful. Instead, the reader needs to see a stain of the canals, the canal pores and/or the canal neuromasts. To show the canals and the pores, one could simply fill the canal with methylene blue to visualize its course. The pores and the canal neuromasts normally also are stained in a DASPEI stain, but in Fig. 8A I do not see them. But it is necessary for your conclusions to show that indeed there are canal neuromasts in that region.*

*Line 482, figure legend Fig. 9: “Lateral line stimulus”? You may also point out here, that the Y-axis is plotted reversed (from negative to positive values). Figures 8-9: At some point you should explicitly write, how you come from the data shown in Fig. 8B to the curves shown in Fig. 9A. I assume that you plotted normalized pressure along the putative canal neuromasts (dots in Fig. 8B)? But you do not explain this. Help the reader here. Figure 9: I compared that curves in Fig. 9A with those in Fig. 7 by Windsor et al (2010a). While the blue curve seems to be the curve from the Hassan-data. The green curve seems to be a curve that is derived from a NACA airfoil. The actual 2D fish data look different. Check red curve in Fig. 7 by Windsor et al). It actually does have two negative peaks but the positive peak in between is very much smaller than the one here in S. rhinoceros. Also, I recommend to label all curves with the species name (A. mexicanus in both cases) and give the respective references in parentheses).*

**Response:**

Line 369: We corrected the text [Lines 349-351]. We deleted the word “more” and added the phrase “seemingly randomly”.

Line 403: On [Line 378] we have changed “showed” to “shows”.

Line 403ff: Fig 7A shows an example, and the drag coefficient of this example is 0.074 that can be derived from the slope coefficient. [Lines 379-380] We added that the data in Figure 7A generate a drag coefficient of 0.074 to the text.

Line 407: The Re in experiment is estimated by the average swimming speed that is fixed. [Lines 381-383] We added that the same Re value is assumed for the experiment.

Lines 408, 409, 410/411: We have revised the text as suggested. Please see details in [Lines 407-411].

Line 422: We changed “indicated” to “indicates” in the legend of Fig 7.

Line 428: We added “lateral line” to the phrase “sensing distance.”

Line 429: We have revised the text about velocity, it, please see details in [Line 415-418]. The revised text is “Finally, as the fish exited the laser plane, $E_{L}$ decreased and the water in the laser plane gradually resumed its initial undisturbed state as the *S. rhinocerous* individual swam far enough away from the laser plane such that the water velocity eventually decreased to values similar to stage 1.”

Line 443ff: We attempted to follow the reviewer’s suggestion for Fig. 8C, but we were unable to make it clear because the outline of the fish shows up as an irregular ellipse that is confusing to the reader.

Line 450: We have revised the “lateral stimulus (CFD)” to “lateral line stimulus (CFD)”.

Line 451: We separated the Fig. 8 into a new Fig. 2 and a new Fig. 9. In Fig 9, the canal pores were assumed to be spaced at 2% body length (BL) intervals as illustrated on the scanned model cavefish body. This pore interval is consistent with an earlier study of Mexican cave characins (Windsor et al., 2010a).

Line 482: We added the “Lateral line stimulus” to Fig 10B. We also noted that the “the Y-axis is plotted reversed” in the legend of Fig 10. We have explained that Fig 10A shows the normalized pressure along the putative canal neuromasts (dots in Fig. 9) in the text. [Lines 448-449] Text now reads: Fig 10A shows the normalized pressure along the putative canal neuromasts in Fig 9.” We also labeled the green curve is derived from NACA0013 3D which was used to represent *Astyanax Mexicanus* (Windsor et al., 2010a) in Fig 10.

Fig 10. **Lateral line stimulus estimation.** CFD results showing (A) the normalized pressure, or *C_p_*, and (B) ${\Delta C}_{p}$ values along the assumed line of canal neuromasts for the simulations. Results extracted from earlier studies are also included. The Y-axis is plotted reversed.

**Comments on discussion:**

*Line 495-496: The sentence reads odd. Maybe you need to shorten this to “Understanding the …. may help to discern …”*

*Line 509: Unclear. Do you mean that the wall-following phase is the exploratory phase?*

*Line 525: Delete text in parenthesis. You explain the methods a few lines further below.*

*Line 545-546: slow swimming may improve sensory capabilities. Why? Give a rational or a logic why this is so.*

*Line 553: It is conjectured. Do you want to say“We therefore hypothesize…”?*

*Line 563: pressure fields matched the locations of the canal pores. Unclear description. Be more explicit. What exactly do you mean?*

*Line 573: “It is conjectured.” Do you want to say “We propose that…”? After all, the data do not and cannot show this.*

*Line 607: Why should this not have a similar function. If the form is similar to the form in S.r., then one would expect similar hydrodynamic effects. And as you hypothesize yourself, better sensing may lead to increased fitness, even if thus a trait only found in males.*

*Lines 656ff: I really think that the future plans can be omitted. By all due respect for your research, but it is really not important for the present paper what you want to do next. If these plans contain arguments relevant for the interpretation of the present data, then these arguments can be discussed in those sections of the Discussion where they are appropriate. If this future research is essential for the data, then maybe one should perform this research now and include it in the paper.*

**Response**

Line 495-496: We have revised this sentence. [Lines 464-466] The text now read: “Understanding the swimming and sensory capabilities of troglobite cavefish may help discern micro-habitat preferences and dispersal patterns of the cavefish in their underground environments.”

Line 509: We added “suggestive of exploratory behavior” to the end of the first sentence to make it clear we are referring to the near-wall-following behavior.

Line 525: We have deleted the text in parenthesis.

Lines 545-546: We changed the sentence to be: In addition, slow swimming behavior may affect the sensory capabilities of the lateral line system and can help to conserve energy.

Line 553: We changed “it is conjectured” to “we therefore hypothesize”.

Line 563: We have revised this sentence. [Lines 528-529] The revised test is: “The simulated pressure value in the location of the canal pores in Fig 9 for *S. rhinocerous* and *S. angustiporus* was extracted.”

Line 573: We changed “conjectured” to “we propose that”.

Line 607: We agree with the comment that the head horn structure of female Kurtus gulliveri may also have the same function on increasing the lateral line stimulus. We revised the text. [Lines 563-574] The new text is: “There are a few non-hypogean fish species that also have a head-horn. For instance, Indo-Pacific surgeonfishes of the genus Naso (Family Acanthuridae); however, it is unlikely that the same function of Naso head-horn is necessary. Because *Naso* differs from *S. rhinocrous* in eyesight and superficial neuromast distribution. *Naso* keep eyesight and they don’t show extensive array of superficial neuromasts. Another example is the nurseryfishes of Australia and Asia (Family Kurtidae; Kurtus gulliveri and Kurtus indicus, respectively), …”

Line 656ff: We have kept the Future Plans Part to discuss the influence of only one sample used in this study and as way to synthesize what we found in the context of how next to reduce uncertainties.
